# Mid‐term patient‐reported outcomes are inferior in opening‐wedge high tibial osteotomy patients with untreated medial meniscus posterior root tear

**DOI:** 10.1002/jeo2.70064

**Published:** 2024-10-21

**Authors:** Eiji Sasaki, Shugo Maeda, Takahiro Tsushima, Yuka Kimura, Yukiko Sakamoto, Eiichi Tsuda, Yasuyuki Ishibashi

**Affiliations:** ^1^ Department of Orthopaedic Surgery Hirosaki University Graduate School of Medicine Hirosaki Japan; ^2^ Department of Orthopaedic Surgery Aomori Rosai Hospital Hachinohe Japan; ^3^ Department of Rehabilitation Medicine Hirosaki University Graduate School of Medicine Hirosaki Japan

**Keywords:** high tibial osteotomy, medial meniscus posterior root tear, mid‐term outcome, patient‐reported outcome, untreated

## Abstract

**Purpose:**

The impact of untreated medial meniscus posterior root (MMPR) tear (MMPRT) during opening‐wedge high tibial osteotomy (OWHTO) on patient‐reported outcomes (PROs) remains poorly understood. This retrospective cohort study aimed to investigate the association between the presence of MMPRT and post‐operative PROs in patients who underwent OWHTO.

**Methods:**

A total of 83 knees that underwent OWHTO that were followed up for 6.6 years were included. Post‐operative PROs were assessed using the knee injury and osteoarthritis outcome score (KOOS) subscales. Medial meniscus extrusion (MME) was measured by magnetic resonance imaging (MRI). MMPRT was diagnosed based on preoperative MRI and intraoperative arthroscopy findings. The participants were categorized into the MMPRT and MMPR intact (MMPRI) groups, and their KOOS subscales were compared. Additionally, logistic regression analysis was conducted to explore the correlation between KOOS and MMPRT presence.

**Results:**

In total, 29 out of 80 (36.3%) knees were classified into the MMPRT group, while three knees underwent total knee arthroplasty. Preoperative MME was 3.5 ± 1.9 (range 0–8.9) mm, showing correlation with the presence of MMPRT (*p* = 0.004) by regression analysis. The post‐operative KOOS subscales of the MMPRT group were lower than the MMPRI group for pain (*p* = 0.017), activities of daily living (ADLs) (*p* = 0.001), sports (*p* < 0.001) and quality of life (QOL) (*p* < 0.001). Additionally, regression analysis showed the presence of MMPRT was correlated with lower KOOS subscale scores for pain (*p* = 0.041), ADLs (p = 0.011), sports (*p* < 0.001) and QOL (*p* = 0.002).

**Conclusion:**

Preoperative MMPRT correlated with a reduction in mid‐term post‐operative PROs, as assessed using the KOOS, among patients who underwent OWHTO. Surgeons should consider addressing an MMPRT at the time of OWHTO.

**Level of Evidence:**

Level IV.

**Abbreviations**
ADL: activity of daily living
ICRS: International Cartilage Research Society
JLCA: joint line convergence angle
KOOS: knee injury and osteoarthritis outcome scales
MME: medial meniscus extrusion
MMPR: medial meniscus posterior root
MMPRT: medial meniscus posterior root tear
MPTA: medial proximal tibia angle
OA: osteoarthritis
OWHTO: opening‐wedge high tibial osteotomy
PRO: patient‐reported outcome
PTS: posterior tibia slope
QOL: quality of life
WBLR: weight‐bearing line ratio

## INTRODUCTION

The management of meniscal lesions in patients with unicompartmental knee osteoarthritis (OA) lacks established clinical guidelines. While repairing the medial meniscus during opening‐wedge high tibial osteotomy (OWHTO) can restore meniscal function and alleviate femorotibial contact pressure [28], controversy surrounds the necessity of repairing medial meniscus posterior root tears (MMPRTs). The prevalence of MMPRT ranges from 10.1% to 27.8% [4, 26], and it is recognized as one of the definitive risk factors for the progression of knee OA or subchondral bone insufficiency fractures due to excessive contact pressure on the medial femorotibial joint [1, 31].

Although acute MMPRT in well‐aligned young patients is a good indication for repair [4], the healing rate on second‐look arthroscopy is sub‐optimal for middle‐aged patients with mild knee OA [27, 34]. Despite this, biomechanical tests have revealed that MMPRT repair during OWHTO restores meniscal function and reduces femorotibial contact pressure [28]. However, OWHTO itself yields favourable clinical outcomes even in knees with meniscal deficits [30]. Furthermore, the clinical and radiographic outcomes are not affected by MMPRT repair or resection during OWHTO [18]. Consequently, the necessity of MMPRT repair during OWHTO and its influence on post‐operative patient‐reported outcomes (PROs) remains uncertain.

This study aimed to investigate the relationship between MMPRT and post‐operative PROs in patients who have undergone OWHTO. We hypothesized that the PROs of patients with MMPRT are inferior to patients without MMPRT even after OWHTO.

## METHODS

### Patients

**Figure 1:**
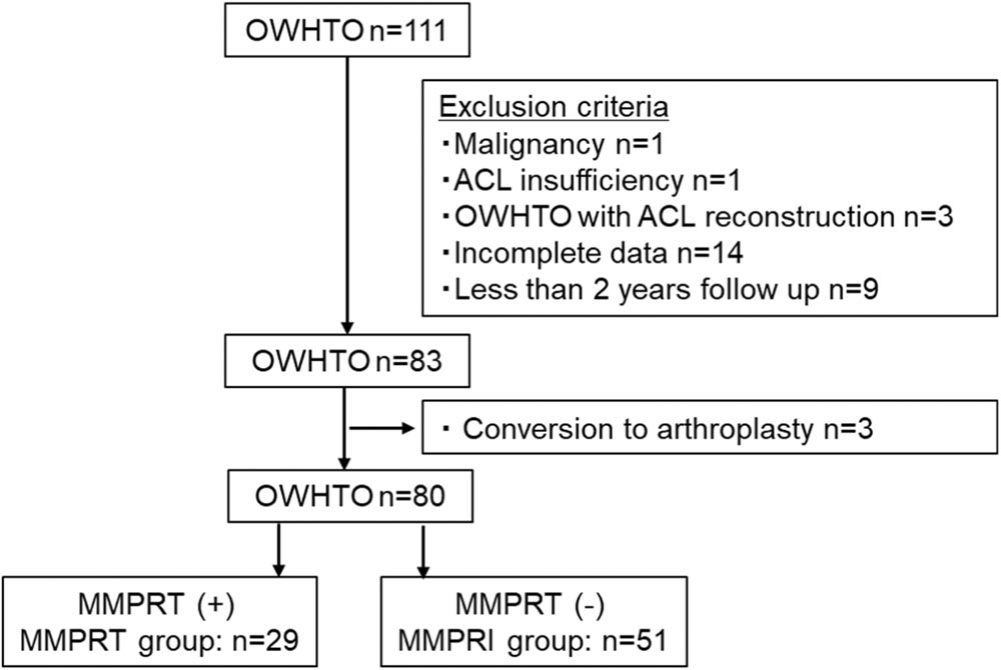
Patients recruitment flow. ACL, anterior cruciate ligament; MMPRI, medial meniscus posterior root intact; MMPRT, medial meniscus posterior root tear; OWHTO, open wedge high tibial osteotomy.

This retrospective cohort study included 111 consecutive knees that underwent medial OWHTO for medial unicompartmental knee OA by multiple surgeons in our hospital between November 2008 and June 2019 (Figure 1), and they were followed up for a minimum of 2 years. The inclusion criteria for OWHTO were symptomatic varus knee OA accompanied by articular cartilage lesions with or without subchondral bone insufficiency fracture of the knee on the medial femoral condyle or medial tibial plateau, with varus alignment as the weight‐bearing line passing through less than 40% of the width of the tibial plateau in relation to the medial end regardless of previous partial meniscectomy and age. The exclusion criteria were active knee infection (*n* = 0); inflammatory diseases, such as rheumatoid arthritis (*n* = 0), malignancy (*n* = 1), severe OA of the patellofemoral or lateral femorotibial joints with Kellgren–Lawrence Grade 3 and over (*n* = 0), Kellgren–Lawrence Grade 4 medial femorotibial OA (*n* = 0); flexion contracture greater than 15° (*n* = 0), anterior cruciate ligament (ACL) insufficiency (*n* = 1), OWHTO with ACL reconstruction (*n* = 3); incomplete data regarding self‐reported questionnaires and radiographs (*n* = 14); and less than 2 years follow up (*n* = 9). A total of 83 out of 111 knees were included. During post‐operative follow‐up, three patients underwent total knee arthroplasty, and 80 knees were included in the statistical analysis. Demographic data, including age, sex and body mass index (BMI) of all patients at the time of surgery and range of motion of the knee evaluated by goniometer were collected from medical records.

### Arthroscopic evaluation of cartilage and meniscal lesions

Arthroscopy was performed before OWHTO to assess the conditions of the cartilage, meniscus, and anterior and posterior cruciate ligament. The severity of articular cartilage damage was scored from 0 to 4 according to the International Cartilage Research Society (ICRS) grading system [19] on the medial and lateral femoral condyle, medial and lateral tibial plateau, trochlea, and patella. Superficial lesions and cracks or fissures were considered ICRS Grade 1, whereas lesions extending less than 50% of cartilage depth were considered ICRS Grade 2. Lesions that did not include the subchondral bone but extended more than 50% of the cartilage depth were considered ICRS Grade 3. Defects that included the subchondral bone were classified as ICRS Grade 4. The total ICRS scores of the six locations were used for statistical analysis as an index of degeneration.

The condition of the meniscus was evaluated through arthroscopy, and MMPRT was diagnosed when the medial meniscal posterior root was completely detached from the tibia or continued with only fibrous tissue with instability upon probing.

### Surgical procedure

Before the surgery, the target for the post‐operative lower limb alignment was planned to align the body weight line passing through the Fujisawa point, which represents 62.5% of the width of the tibial plateau relative to the medial end [6, 24]. Digital planning software (MediCAD, Hectec GmbH) was utilized for planning from an anteroposterior (AP) view on whole‐leg radiographs under full weight‐bearing conditions.

Following arthroscopy, OWHTO was performed using various plates: the Puddu plate (Arthrex®), the Position HTO plate (Aesculap®) and the Tris Medial HTO Plate System (OLYMPUS®). A longitudinal skin incision was made on the medial aspect of the proximal tibia, and the pes anserinus was identified. The first layer was incised along the superior border of the pes anserinus and the pes anserinus was retracted posteriorly to expose the superficial medial collateral ligament. The osteotomy level was determined by fluoroscopy, and the superficial medial collateral ligament was transected using a chisel and knife. Monoplane or biplane osteotomy was performed, leaving the lateral 10 mm of the tibial head intact as a hinge for osteotomy [37]. The weight‐bearing line ratio (WBLR) was measured from the centre of the femoral head to the talus using an alignment rod under fluoroscopic guidance. While the opening gap was determined by the WBLR based on preoperative planning, it was reduced in preoperative planning when the medial proximal tibial angle exceeded 95°. The wedged β‐tricalcium phosphate block (HOYA Technosurgical Co. Ltd.) was placed posteriorly in the opening gap space, and the proximal tibia was fixed to each plate. As post‐operative rehabilitation, patients were allowed partial weight bearing immediately after surgery for 3 weeks, then full weight bearing was allowed. Range of motion exercises was started post‐operative Day 1. Depending on the post‐operative pain, patients were allowed to return to their daily activity, and full activities were allowed after bony union of the osteotomy site.

### Diagnosis of MMPRT

**Figure 2:**
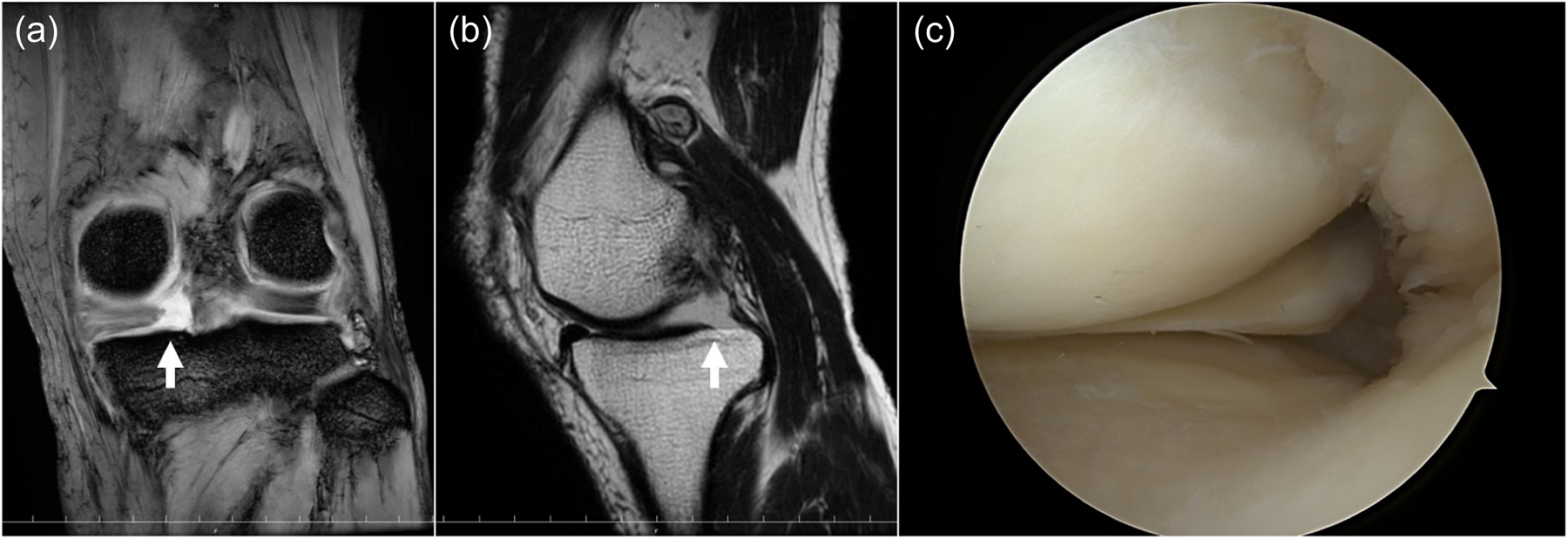
MMPRTs MRI findings of MMPRT are shown. Giraffe neck sign in coronal view (a) and ghost sign in sagittal view (b) are shown. Also, arthroscopy revealed discontinuity of MMPRT (c). MMPRT, medial meniscus posterior root tear; MRI, magnetic resonance imaging.

MMPRT was retrospectively diagnosed on magnetic resonance imaging (MRI; Figure 2a,b) or arthroscopy (Figure 2c), and then patients were classified into MMPR intact (MMPRI) and MMPRT groups. MRI revealed that the white meniscus, ghost, and cleft signs were positive for MMPRT. Medial meniscus extrusion (MME) was measured in the coronal plane [38]; it was defined as the tangential distance from the cortical bone on the medial side of the tibia to the outer border of the medial meniscus.

### Evaluation of PROs

Post‐operative PROs related to knee symptoms were evaluated using the knee injury and osteoarthritis outcome score (KOOS) at their final follow‐up, comprising of 42 knee‐related items, with each item being scored from 0 to 4 [32]. Total scores on five subscales (symptoms, pain, activities of daily living [ADLs], sports/recreation and quality of life [QOL]) were converted to 100 points as the best conditions. The KOOS results were evaluated on the operative side without considering the contralateral side.

### Radiographic evaluation

Preoperative and post‐operative radiographic parameters were measured using the digital planning software (MediCAD, Hectec GmbH) by two senior orthopaedic surgeons, separately. Post‐operative radiographs were obtained at the 1‐year follow‐up after OWHTO. The WBLR, joint line convergence angle (JLCA) and medial proximal tibial angle (MPTA) were measured using the anteroposterior views of the whole leg under weight‐bearing conditions. The mechanical axis was defined as the line from the centre of the femoral head to the centre of the talus on a weight‐bearing radiograph. The WBLR was defined as the percentage of the width of the tibial plateau and the mechanical axis passed relative to the medial end [12]. The MPTA was defined as the medial angle formed by the mechanical tibial axis and a line tangential to the joint surface of the proximal tibial plateau. The posterior tibia slope (PTS) was measured in the lateral view of knee radiographs [10]. The second measurement was performed one month after the first measurement, and intra‐observer reliability was calculated. The intra‐ and intra‐observer reliabilities of these parameters were 0.872–0.970 and 0.820–0.970, respectively.

### Statistical analysis

To achieve 80% statistical power with an alpha of 0.05, power analysis revealed that a minimum of 62 patients would be required to detect differences in KOOS QOL between the MMPRI and MMPRT groups. In the post hoc analysis, the statistical power of 80 patients was calculated to be 0.886, with an effect size of 0.746.

Quantitative data are expressed as mean ± standard deviation. The chi‐square test was used to evaluate differences in categorical variables, and the Mann–Whitney *U* test was used to evaluate differences in continuous variables between the MMPRI and MMPRT groups. To analyse the factors related to the presence of MMPRT, logistic regression analysis was performed with MMPRT as the dependent variable and age, sex, radiographic parameters, MME and the summed ICRS score as the independent variables. The KOOS subscales between the MMPRI and MMPRT groups were compared using the Mann–Whitney *U* test. To investigate the factors related to the post‐operative KOOS subscales, multiple linear regression analyses were performed with the KOOS subscales as dependent variables and age, sex, preoperative BMI, preoperative MMPRT, post‐operative knee extension and flexion angles, JLCA, MPTA and PTS as the independent variables. These regression models were adjusted according to the follow‐up period. Data input and analysis were performed using the SPSS software (version 27.0; SPSS Inc.). Statistical significance was set at *p* < 0.05.

## RESULTS

The mean follow‐up period was 6.6 ± 3.0 (2.0–12.8) years, during which 3 out of 83 (3.6%) knees underwent total knee replacement. A total of 29 out of 80 (36.3%) knees were classified into the MMPRT group, with a higher prevalence of MMPRT in women (51.2%) than in men (*p* = 0.003; Table 1). Mean age was 55.1 ± 9.2 years old, and BMI was 25.9 ± 3.4 kg/m^2^. Mean preoperative values of WBLR, JLCA, MPTA and PTS were 22.3 ± 13.8%, 3.1 ± 1.6°, 84.3 ± 2.9° and 8.5 ± 3.0°, respectively. In addition, the final JLCA, MPTA and PTS values were 2.0 ± 1.4°, 89.6 ± 2.0° and 9.7 ± 2.6°, respectively. The mean MME of the MMPRT group was 5.4 ± 1.1 mm, significantly larger than 2.4 ± 1.3 mm of the MMPRI group (*p* < 0.001). Regression analysis revealed that radiographic parameters were not associated with the presence of MMPRT, except for a large MME (*p* = 0.004; Table 2).

**Table 1:** Patients' preoperative demographics and radiographic parameters.

	MMPRI		MMPRT	
	Preoperative	Final	Preoperative	Final
Sample number	51		29	
Age at surgery, years	54.0 ± 10.2		57.0 ± 6.8	
Women, %	21 (41.2%)		22 (75.9%)	
Body mass index, kg/m^2^	25.5 ± 3.1		26.6 ± 3.8	
Follow‐up period, years	6.1 ± 2.8		7.5 ± 3.2	
Knee extension angle, °	−2.4 ± 4.0	−1.5 ± 3.3	−2.5 ± 3.6	−3.0 ± 5.1
Knee flexion angle, °	138.1 ± 7.3	140.9 ± 7.3	137.8 ± 7.7	136.4 ± 7.9
BWLR, %	24.5 ± 13.7	–	18.1 ± 13.2	–
JLCA, °	2.5 ± 1.2	1.8 ± 1.4	4.1 ± 1.7	2.3 ± 1.5
MPTA, °	84.3 ± 3.3	89.4 ± 2.2	84.4 ± 2.2	90.0 ± 1.4
PTS, °	8.8 ± 3.1	9.5 ± 2.4	7.9 ± 2.8	10.0 ± 3.0
MME, mm	2.4 ± 1.3	–	5.4 ± 1.1	–
Total ICRS score	9.5 ± 4.8	–	9.9 ± 3.8	–

*Note*: Values indicate mean ± standard deviations of demographic data and imaging parameters of the MMPRI and MMPRT groups that were compared using the Mann–Whitney *U* test or chi‐square test.

Abbreviations: ICRS, International Cartilage Repair Society; JLCA, joint‐line convergence angle; MME, medial meniscus extrusion; MMPRI, medial meniscus posterior root intact; MMPRT, medial meniscus posterior root tear; MPTA, medial proximal tibial angle; PTS, posterior tibial slope; WBLR, weight‐bearing line ratio.

**Table 2:** Preoperative factors related to the presence of MMPRT.

	*B*	*p*	Odds ratio	95% CI
Age at surgery	0.13	0.273	1.14	0.90–1.44
Women	1.60	0.276	4.93	0.28–86.96
Body mass index	0.22	0.391	1.24	0.76–2.04
Preoperative WBLR	−0.01	0.851	0.99	0.86–1.13
Preoperative JLCA	0.16	0.705	1.18	0.51–2.70
Preoperative MPTA	−0.11	0.798	0.90	0.39–2.05
Preoperative PTS	−0.49	0.065	0.61	0.36–1.03
Preoperative MME	**3.04**	**0.002**	**20.97**	**2.96–148.58**
Total ICRS scores	−0.04	0.800	0.96	0.69–1.33

*Note*: To identify factors related to the preoperative presence of MMPRT, logistic regression analysis was performed with the presence of MMPRT as the dependent variable, age, sex, radiographic parameters, degree of medial meniscus extrusion (MME), and the summed ICRS score as the independent variables.

Abbreviations: B, correlation coefficient; JLCA, joint line convergence angle; MMPRT, medial meniscus posterior root tear; MPTA, medial proximal tibial angle; PTS, posterior tibial slope; WBLR, weight‐bearing line ratio; 95% CI, 95% confidence interval.

**Figure 3:**
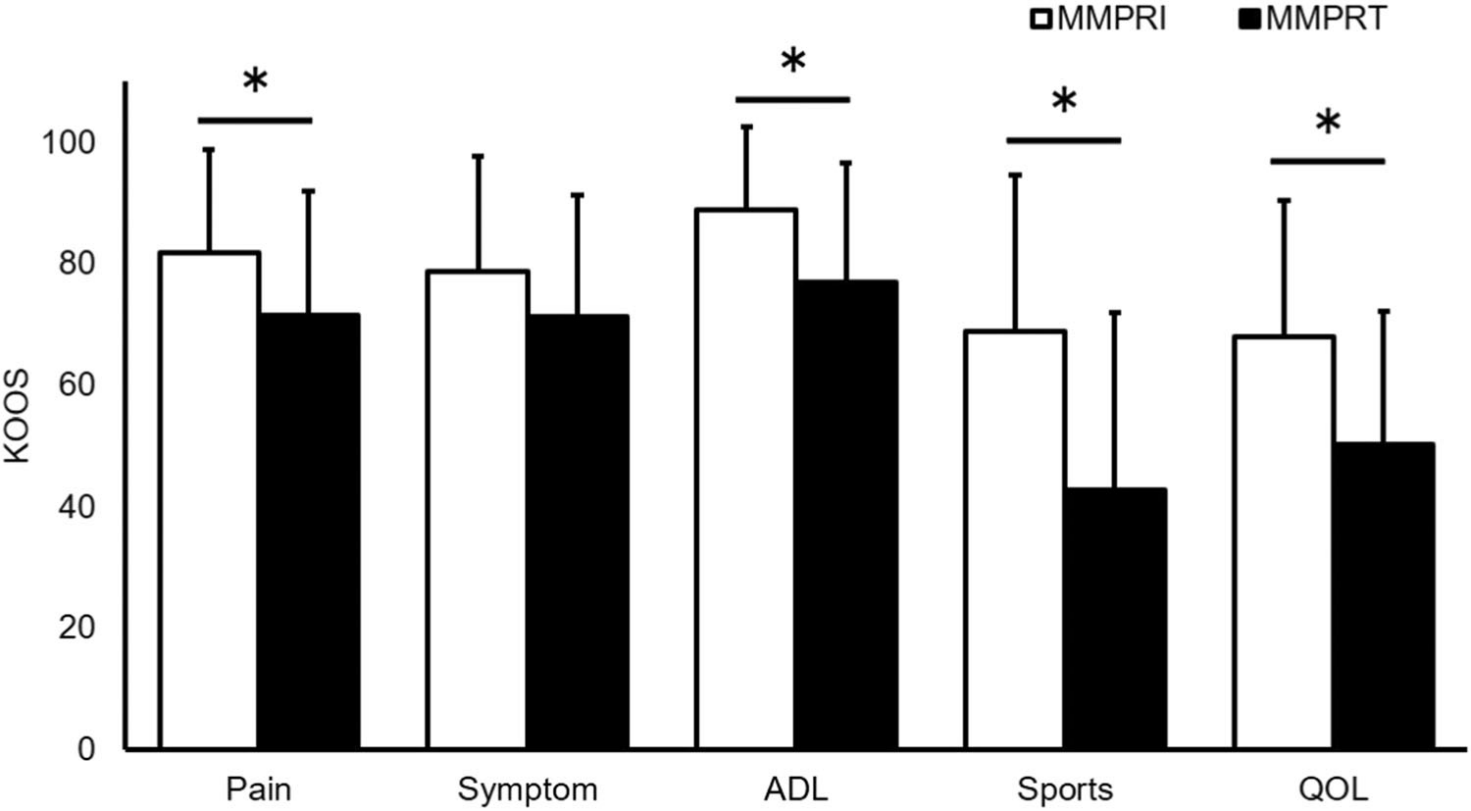
Comparison of the KOOS subscale scores in patients with and without untreated MMPRT. The KOOS subscale scores between the MMPRI and MMPRT groups were compared using the Mann–Whitney *U* test. A *p* value below 0.05 was considered significant (*). ADL, activity of daily living; KOOS, knee injury and osteoarthritis outcome score; MMPRI, medial meniscus posterior root intact; MMPRT, medial meniscus posterior root tear; QOL, quality of life.

The KOOS subscales of the MMPRT group were as follows: pain, 71.4 ± 20.6; symptoms, 71.2 ± 20.1; ADLs, 76.9 ± 19.5; sports/recreation, 42.7 ± 29.2 and QOL, 50.2 ± 21.9 (Figure 3). Compared to the MMPRI group, the MMPRT group had lower scores in four subscales: pain, 81.8 ± 16.9 (*p* = 0.017); symptoms, 78.6 ± 19.0 (*p* = 0.077); ADLs, 88.8 ± 13.7 (*p* = 0.001); sports/recreation, 68.7 ± 25.8 (*p* < 0.001) and QOL, 67.9 ± 22.4 (*p* < 0.001). Another regression analysis revealed that the presence of MMPRT was correlated with lower KOOS for pain (*p* = 0.041), ADLs (*p* = 0.011), sports (*p* < 0.001) and QOL (*p* = 0.002) at the final follow‐up (Table 3). Additionally, KOOS for ADLs (*p* = 0.048) and QOL (*p* = 0.044) were negatively associated with BMI, while those for symptoms (*p* = 0.007) and sports/recreation (*p* = 0.031) were positively correlated with the knee flexion angle.

**Table 3:** Related factors for post‐operative patient‐reported outcomes.

	Pain		Symptom		ADLs		Sports		QOL	
	*β*	*p*	*β*	*p*	*β*	*p*	*β*	*p*	*β*	*p*
Age at surgery	−0.20	0.165	0.04	0.751	−0.18	0.193	−0.06	0.634	−0.25	0.052
Women	−0.10	0.444	−0.19	0.126	−0.19	0.117	−0.15	0.198	−0.08	0.476
Preoperative body mass index	−0.12	0.369	0.01	0.976	**−0.25**	**0.048**	0.01	0.946	**−0.24**	**0.044**
Preoperative MMPRT	**−0.26**	**0.041**	0.35	0.129	**0.19**	**0.011**	**0.15**	**<0.001**	**0.27**	**0.002**
Follow‐up period	**0.26**	**0.046**	**−0.19**	**0.006**	−0.32	0.119	**−0.44**	0.214	**−0.37**	**0.024**
Final knee extension angle	0.06	0.648	0.06	0.604	0.06	0.586	0.08	0.473	0.01	0.926
Final knee flexion angle	0.13	0.326	**0.36**	**0.007**	0.01	0.928	**0.27**	**0.031**	0.09	0.465
Final JLCA	−0.07	0.608	−0.04	0.763	0.01	0.944	0.02	0.864	0.01	0.931
Final MPTA	0.06	0.679	−0.07	0.594	−0.03	0.831	−0.04	0.747	0.02	0.864
Final PTS	−0.09	0.484	−0.07	0.564	−0.06	0.651	−0.03	0.798	−0.07	0.559

*Note*: To identify related factors for the five subscales of the KOOS, multiple linear regression analyses were performed with KOOS subscales as dependent variables, age, sex, preoperative BMI, preoperative MMPRT, post‐operative knee extension and flexion angles, JLCA, MPTA and PTS as independent variables. These regression models were adjusted according to the follow‐up period.

Abbreviations: β, standardized correlation coefficient; ADL, activity of daily living; BMI, body mass index; KOOS, knee injury and osteoarthritis outcome score; JLCA, joint line convergence angle; MMPRT, medial meniscus posterior root tear; MPTA, medial proximal tibial angle; PTS, posterior tibial slope; QOL, quality of life.

## DISCUSSION

The primary finding of the present study was that the mid‐term KOOS subscales were inferior in patients with MMPRT at the time of OWHTO compared with patients without MMPRT, with no difference in the summed ICRS score. These findings suggest that repair of MMPRT should be performed in patients with unicompartmental knee OA indicated for OWHTO.

This study revealed that the KOOS subscales were reduced in the presence of MMPRT. Previous studies have suggested that MMPRT has less influence on the clinical outcomes of patients who underwent OWHTO. For instance, Kim et al. reported that in the short‐ to mid‐term follow‐up of patients with MMPRT who underwent OWHTO, their radiological or clinical outcomes were not inferior to patients without MMPRT. Post‐operative lower‐extremity alignment, rather than MMPRT, influenced OWHTO outcomes [17]. Additionally, medial joint space narrowing progresses even after repair of the MMPRT [16]. Regarding post‐operative patient satisfaction, satisfaction rates of 75%–89% were reported [8, 23, 33, 36]. Goshima et al. reported that patient satisfaction after OWHTO was up to 88.6%, and the reasons for dissatisfaction were unmet for preoperative expectations in terms of pain and function [8]. In current cases, dissatisfaction with KOOS pain and sports was similarly observed, which might be influenced by the presence of MMPRT in patients who underwent OWHTO.

Various risk factors for MMPRT or the spreading root sign have been reported, including age, female sex, high BMI, low physical activity, steeper posterior tibial slope, intercondylar notch narrowing, severity of OA, degeneration of the ACL and lower limb alignment [7, 11, 13]. In the current study, only MME was related to MMPRT, regardless of age, female sex, BMI and radiographic parameters. This finding may be attributed to participants with moderate OA who underwent OWHTO, including 36.3% with MMPRT. MME is recognized as one of the easily identifiable imaging findings indicative of medial meniscus hoop dysfunction diagnosed using MRI or ultrasonography [5, 35]. It is also useful in detecting MMPRT in patients scheduled for OWHTO.

Previous reports showed that more than half of the patients with MMPRT undergo nonsurgical treatment, while others opt for arthroscopic treatment, high tibial osteotomy, or arthroplasty [21]. However, findings from a 10‐year observational study revealed that the nonsurgical management of MMPRT led to failure in 95% of patients, with 64% of patients requiring arthroplasty [20]. Moreover, a 6‐year observational study identified nonoperative management or partial meniscectomy for MMPRT as risk factors for arthroplasty compared to MMPRT repair [2]. Therefore, it is advisable to repair MMPRTs whenever possible. However, clinical outcomes of isolated MMPRT repair are unsatisfactory. Second‐look arthroscopy demonstrated that complete healing was not achieved with isolated repair of MMPRT [34]. In contrast, OWHTO improved clinical outcomes, even in knees with meniscal deficits [30]. Additionally, the repair of MMPRT during OWHTO showed a superior healing rate compared to that of unrepaired MMPRT [22]. Hence, combined OWHTO and MMPRT repair should be considered for these patients.

The optimal treatment strategy for MMPRT in OWHTO remains controversial. Partial resection of MMPRT did not impact the outcome of medial OWHTO [18]. In addition, clinical outcomes improved in patients undergoing OWHTO without repair of MMPRT [25]. However, this study revealed that leaving MMPRT untreated during OWHTO led to inferior mid‐term clinical outcomes. There is a general consensus that MMPR repair can restore meniscal hoop tension and fundamentally prevent OA progression [3, 29]. A recent biomechanical study indicated that repairing the MMPRT during OWHTO can restore meniscal function and reduce femorotibial contact pressure, which increases by 180% with MMPRT [28]. When MMPRTs were resected during OWHTO, 41.9% of them exhibited remodelling, with adhesion to the posterior cruciate ligament [18]. However, its efficacy in restoring the hoop function of the medial meniscus has not been validated by biomechanical studies. Various surgical techniques, such as pullout repair [9, 14] or suture to the posterior cruciate ligament [15], have been reported for repairing MMPRTs. Therefore, determining the appropriate approach for combined surgery with OWHTO remains unresolved.

### Limitations

This study has some limitations, primarily due to its retrospective design. First, this retrospective cohort study did not include a repair group, thus limiting our ability to compare outcomes with MMPRT repair. However, upon the completion of this series, we will conduct MMPRT repair, allowing for a historical comparison between MMPRT and MMPR repair groups. Second, the preoperative KOOS was not investigated. Additionally, our study lacked evaluations using other subjective scoring systems, satisfaction scales or activity evaluations. A more comprehensive evaluation would have facilitated a clearer detection of the MMPRT problem. Third, post‐operative WBLR was lacking in this case series. Furthermore, structural changes and the severity of OA were not evaluated. The combined evaluation of symptoms and imaging findings will improve our understanding of post‐operative satisfaction. Despite these limitations, this study revealed that the presence of untreated MMPRT influenced midterm PROs after OWHTO.

## CONCLUSIONS

Preoperative MMPRT correlated with a reduction in mid‐term post‐operative PROs, as assessed by the KOOS, in patients who underwent OWHTO. Given the confirmation of high incidence and substantial impact of MMPRT on post‐operative outcomes, MMPRT in patients with unicompartmental knee OA should not be left untreated at the time of OWHTO.

## AUTHOR CONTRIBUTIONS

All authors were involved in the planning of the study design and in the execution of the study. Eiji Sasaki and Yasuyuki Ishibash handled the analysis of this study and drafted the manuscript. All authors have corrected and approved the final version of the manuscript.

## CONFLICT OF INTEREST STATEMENT

The authors declare no conflict of interest.

## ETHICS STATEMENT

This observational study was approved by the Hirosaki University Graduate School of Medicine's ethics committee (approval number: 2019‐596‐1). Informed consent was obtained from all patients for the treatment and use of their clinical data for research and publication. All participants provided written informed consent, and the study was conducted in accordance with the 1964 Helsinki Declaration (and its later amendments or comparable ethical standards).

## Data Availability

The data sets generated during and/or analyzed during the current study are available from the corresponding author upon reasonable request.
